# Quantitative assessment of the association between erector spinae muscle and in-hospital mortality in elderly patients with pulmonary tuberculosis

**DOI:** 10.1186/s13104-021-05546-3

**Published:** 2021-04-13

**Authors:** Ai Tanaka, Kosaku Komiya, Mari Yamasue, Yumiko Ando, Yukiko Takeno, Shuichi Takikawa, Kazufumi Hiramatsu, Jun-ichi Kadota

**Affiliations:** 1 Department of Internal Medicine, National Hospital Organization Nishi-Beppu Hospital, 4548 Tsurumi, Beppu, Oita 874-0840 Japan; 2 Department of Respiratory Medicine and Infectious Diseases, Oita University Faculty of Medicine, 1-1 Idaigaoka, Hasama-machi, Yufu, Oita 879-5593 Japan; 3 Department of Radiology, National Hospital Organization Nishi-Beppu Hospital, 4548 Tsurumi, , Beppu, Oita 874-0840 Japan

**Keywords:** Tuberculosis, Erector spine muscles, Chest CT, In-hospital death

## Abstract

**Objective:**

Skeletal muscle size is considered a predictor of prognosis in patients with respiratory diseases including *Mycobacterium avium* complex lung disease. However, no research focused on its impact on prognosis in patients with pulmonary tuberculosis (TB). Thus, this study aimed to assess the association between erector spinae muscle (ESM) size and in-hospital mortality among patients with pulmonary TB.

**Results:**

We retrospectively included 258 consecutive patients aged over 65 years old, who were admitted to the hospital for bacteriologically confirmed pulmonary TB, and all underwent chest computed tomography (CT) scan upon admission. The cross-sectional area of the ESM (ESMcsa) was measured at the lower margin of the 12th thoracic vertebra on a single-slice CT scan image and was adjusted according to body surface area (BSA). In total, 71 (28%) patients died during hospitalization. The non-survivor group had a high incidence of respiratory failure and comorbidities and lower hemoglobin and albumin levels, performance status score, and ESMcsa/BSA. Multivariate analysis revealed that low performance status score and hemoglobin and albumin levels, but not ESMcsa/BSA and body mass index, could independently predict in-hospital mortality after adjusting for age and comorbidities. Therefore, ESM size was not associated with in-hospital mortality in patients with pulmonary TB.

## Introduction

Tuberculosis (TB) is a major public health threat worldwide, and the coronavirus disease 2019 pandemic had a negative effect on the early and accurate detection of tuberculosis due to lockdown or restrictions in-hospital visits [1]. To control TB infection in the community and to facilitate early detection of pulmonary TB, effective treatment strategies with anti-TB drugs and other non-pharmaceutical interventions should be established. The identification of prognostic factors can be helpful for not only rigorous prediction but also the consideration of effective interventions.

As for a prognostic factor, recent studies have shown the associations between skeletal muscle size and disease severity and prognosis in several respiratory diseases [2–8]. The erector spinae muscle (ESM) is a major muscle that plays a role in respiration in addition to maintaining an erect posture [9]. ESM size can be easily measured when pulmonary diseases are evaluated on chest CT scan, and the quantification of this muscle may be helpful in predicting disease progression in chronic pulmonary disease (COPD) [2–5], idiopathic pulmonary fibrosis (IPF) [6–8, 10], and *Mycobacterium avium* complex (MAC) lung disease [11, 12]. However, no study has focused on its impact on prognosis in patients with pulmonary TB. Therefore, the current study aimed to assess the association between ESM size and in-hospital mortality among patients with active pulmonary TB.

## Main text

### Methods

#### Patients and study design

This was a retrospective cohort study conducted at National Hospital Organization Nishi-Beppu Hospital, the only institution that can accept patients with smear-positive lung TB in Oita Prefecture, Japan. We included consecutive patients aged over 65 years old with bacteriologically confirmed pulmonary TB using real-time PCR for the detection of Mycobacterium tuberculosis DNA who were admitted to the hospital between January 2013 and December 2016. These patients underwent chest CT scan within 1 month before and after admission, and data on height and weight were recorded to calculate body mass index (BMI) and body surface area (BSA) for the analysis of ESM size. The number of samples was calculated using EpiTools (https://epitools.ausvet.com.au/, two-tailed, α error = 0.05 and power = 0.8). In total, 217 patients were required according to a previous report that evaluated the association between ESMcsa and mortality among patients with MAC [11]. The study protocol was approved by the institutional ethics committee of our institution (approval number: 2-04; approval date: June 29, 2020). The need for informed consent was waived by the committee due to the retrospective design of the study. Information regarding this research was posted at the hospital, with an opt out method. Some patients already participated in previous studies [13–17].

#### Data collection

Patient data—including gender, age, BMI, daily physical activity levels and underlying diseases (diabetes mellitus, cerebrovascular disease, Herat failure, COPD, chronic kidney disease, and hepatic disease), smoking history, laboratory data (white blood cell concentration, hemoglobin, serum albumin, C-reactive protein, liver enzymes, blood urea nitrogen, and creatinine), sputum information (smear grade, results of sputum culture, and time to negative conversion), and presence of respiratory failure—were obtained from medical records. Collection and examination of data are routinely performed when a patient diagnosed with lung TB is admitted to our hospital. Then, we evaluated daily physical activity upon admission using a performance status scale [18]. Respiratory failure was defined as an oxygen saturation of 90% without oxygen therapy upon admission. The primary outcome was all-cause in-hospital mortality. The patients who died in the hospital or those who survived to be discharged were classified as the nonsurvivors or survivors. These data were extracted by two respiratory physicians.

#### Evaluation of chest CT scan findings

A 16-detector row CT scanner (Activision, Toshiba Medical Systems, Tokyo, Japan) was used. Scans were performed using 1.0-mm thick sections of contiguous images from the apex to the lung base. Images were obtained at a window setting of –600 HU (level) and 1600 HU (width). If the patient underwent CT scan before referral to our hospital, the CT scan features were evaluated using the images obtained at the referring institutions.

Chest CT images reconstructed using the mediastinal setting were used for the quantitative analysis of the ESM. Using the evaluation method utilized in previous studies [5, 19], the ESM area on cross-sectional CT scan image (ESMcsa) was measured at the lower margin of the 12th thoracic vertebra using the SYNAPSE volume analyzer (FUJIFILM Medical Co., Ltd., Tokyo, Japan). In brief, the left and right ESMs were identified and manually shaded. Moreover, the cross-sectional areas of both ESMs were calculated using NOBORI viewer (TECHMATRI, Tokyo, Japan), a medical information platform, by a respiratory medicine specialist (AT) and a radiologist (YA) who were blinded to the clinical information. The ESMcsa was presented as the sum of the right and left muscles. ESMcsa was adjusted according to BSA (calculated using height and weight).

#### Statistical analysis

Statistical analyses were performed using the Statistical Package for the Social Sciences software version 24 (IBM Japan, Tokyo, Japan). For two-tailed analyses, 95% confidence intervals were calculated. Variables among patients’ backgrounds, laboratory data, presence of respiratory failure, and ESMcsa/BSA with a *P* value of < 0.05 in the univariate analysis were included in the multivariate analysis. To explain why ESMcsa was independent of other variables when used as a predictor of mortality, Cox proportional hazards regression analysis was performed to evaluate the effect of ESMcsa/BSA on in-hospital mortality.

### Results

#### Baseline characteristics of the survivor and non-survivor groups

In total, 262 patients were admitted to the hospital, and all underwent chest CT scan. Among them, 258 (98%) whose height and weight were measured upon admission were eventually included in this study. Approximately 50% of patients were women, and the median patients’ age was 84 (interquartile range: 79–88) years. *M. tuberculosis* that is resistant to more than one first-line anti-TB drug was isolated in 15 (6%) patients. However, no bacterium was resistant to combined isoniazid and rifampin. In total, 71 (28%) patients died during hospitalization. Compared with the survivor group, the non-survivor group had a significantly lower performance status score and hemoglobin and albumin levels; higher incidence of comorbidities, such as chronic kidney diseases, heart failure, and hepatic diseases, and respiratory failure; and greater C-reactive protein (CRP) levels, as shown in Table 1. The ESM_CSA_/BSA of the non-survivor group was significantly lower than that of the survivor group.

**Table 1:** Univariate analysis of the baseline characteristics associated with in-hospital mortality of the patients with pulmonary tuberculosis

	Non-survivor (n = 71)	Survivor (n = 187)	HR	95% CI	*P*
Female	36 (50.7)	94 (50.3)	0.813	0.508–1.302	0.389
Age (y)	87 (81–90)	82 (78–88)	1.036	0.997–1.077	0.073
BMI (kg/m^2^)	17.8 (15.7–20.3)	19.1 (17.4–21.2)	0.944	0.876–1.018	0.133
PS	4 (3–4)	2 (1–3)	2.441	1.745–3.415	< 0.001
DM	19 (26.8)	45 (24.1)	1.091	0.644–1.848	0.745
CVD	14 (19.7)	30 (16.0)	1.164	0.648–2.090	0.612
Heart failure	21 (29.6)	26 (13.9)	1.896	1.135–3.167	0.014
COPD	7 (9.9)	14 (7.5)	1.502	0.685–3.293	0.310
CKD	17 (23.9)	18 (9.6)	2.880	1.658–5.001	< 0.001
Hepatic disease	11 (15.5)	8 (4.3)	3.289	1.712–6.321	< 0.001
Smoker	15 (21.1)	32 (17.1)	1.416	0.799–2.511	0.234
Respiratory failure	43 (60.6)	39 (20.9)	2.882	1.782–4.660	< 0.001
WBC (× 10^3^/µL)	6.6 (4.8–10.0)	6.5 (5.2–8.1)	1.026	0.957–1.100	0.474
Neu (× 10^3^/µL)	5.4 (4.1–8.9)	4.8 (3.6–6.6)	1.074	1.003–1.150	0.040
Hb (g/dL)	10.3 (9.1–11.5)	11.5 (10.2–12.9)	0.722	0.631–0.827	< 0.001
Albumin (g/dL)	2.1 (1.7–2.5)	3.0 (2.5–3.5)	0.203	0.128–0.321	< 0.001
CRP (mg/dL)	6.6 (3.4–12.2)	2.6 (0.8–6.4)	1.057	1.031–1.084	< 0.001
AST (IU/L)	27.0 (20.0–42.5)	25.0 (20.0–35.0)	1.006	1.003–1.009	< 0.001
ALT (IU/L)	17.0 (12.0–27.5)	16.0 (11.0–25.0)	1.006	1.002–1.011	0.004
BUN (mg/dL)	26.0 (17.0–37.3)	16.5 (12.6–21.2)	1.023	1.011–1.034	< 0.001
Cr (mg/dL)	0.76 (0.43–1.10)	0.72 (0.56–0.89)	1.533	1.203–1.954	0.001
eGFR (mL/min/1.73 m^2^)	76.2 (47.7–138.7)	78.4 (61.6–107.1)	1.001	0.997–1.004	0.724
Smear Grade	1 (0–3)	1 (0–3)	1.222	0.812–1.838	0.337
Time to negative conversion (day)	38 (20–53)	48 (32–66)	0.978	0.968–0.988	< 0.001
ESM_CSA_ (mm^2^)	1306.2 (1042.3–1624.4)	1715.7 (1296.2–2328.0)	0.999	0.999–1.000	< 0.001
ESM_CSA_/BSA (mm^2^)	999.7 (784.8–1139.3)	1290.9 (975.0–1558.5)	0.999	0.998–0.999	< 0.001
ESM-CT number (HU)	19.6 (− 3.9–35.5)	28.9 (8.6–40.2)	0.995	0.986–1.003	0.226

Data are presented as the number (%) or median (interquartile range)

BMI: body mass index; PS: performance status; DM: diabetes mellitus; CVD: cerebrovascular disease; COPD: chronic obstructive pulmonary disease; CKD: chronic kidney disease; ESM_CSA_: the cross-sectional area of erector spinae muscles; BSA: body surface area

#### Predictors of in-hospital mortality in patients with pulmonary tuberculosis

We conducted a multivariate analysis in five models because significant differences were observed among similar variables reflecting nutritional or physical status, performance status, hemoglobin and albumin levels, and ESM_CSA_/BSA in the univariate analysis. Results showed that performance status and hemoglobin and albumin levels, but not ESMcsa/BSA and BMI, were significantly associated with in-hospital mortality after adjusting for age, comorbidities, CRP level, and respiratory failure (Table 2).

**Table 2:** Multivariate analysis of the baseline characteristics associated with in-hospital mortality of the patients with pulmonary tuberculosis

	Model 1 (BMI)			Model 2 (PS)			Model 3 (Hb)			Model 4 (Alb)			Model 5 (ESM_CSA_/BSA)		
	HR	95% CI	*P*	HR	95% CI	*P*	HR	95% CI	*P*	HR	95%CI	*P*	HR	95%CI	*P*
Age	1.037	0.996–1.080	0.081	1.013	0.972–1.057	0.539	1.034	0.991–1.079	0.126	1.033	0.985–1.084	0.180	1.019	0.974–1.066	0.408
Heart failure	1.541	0.904–2.628	0.112	1.392	0.819–2.369	0.222	1.523	0.897–2.584	0.119	1.714	0.968–3.035	0.065	1.536	0.906–2.606	0.111
Respiratory failure	2.452	1.386–4.337	0.002	1.883	1.040–3.409	0.037	2.339	1.322–4.139	0.004	1.312	0.705–2.441	0.391	2.407	1.358–4.266	0.003
CKD	4.289	2.339–7.865	< 0.001	3.573	1.936–6.592	< 0.001	4.013	2.170–7.422	< 0.001	4.840	2.522–9.286	< 0.001	4.301	2.333–7.929	< 0.001
CRP	1.045	1.012–1.079	0.007	1.032	1.001–1.063	0.046	1.028	0.996–1.061	0.082	1.003	0.964–1.043	0.879	1.036	1.005–1.067	0.022
AST	1.006	1.003–1.010	< 0.001	1.006	1.002–1.009	0.001	1.007	1.004–1.010	< 0.001	1.004	1.000–1.007	0.050	1.006	1.002–1.009	0.001
BMI	0.964	0.889–1.044	0.366												
PS				1.712	1.177–2.490	0.005									
Hb							0.763	0.660–0.882	< 0.001						
ALB										0.233	0.129–0.421	< 0.001			
ESM_CSA_/BSA													0.999	0.999–1.000	0.127

BMI: body mass index; PS: performance status; Alb: albumin; ESM_CSA_: the cross-sectional area of erector spinae muscles; BSA: body surface area; CKD: chronic kidney disease

### Discussion

This study showed that ESMcsa/BSA was not significantly associated with in-hospital mortality after adjusting for age, comorbidities, CRP level, and respiratory failure in patients with active pulmonary TB. In contrast, physical activity level assessed based on performance status and hemoglobin and albumin levels were significantly correlated with mortality. This result is consistent with that of previous studies showing that these factors play a significant role in predicting poor prognosis [14, 20, 21].

TB and non-TB *Mycobacterium* are bacteriologically under the same category as acid fast bacterium. However, ESM size could be a predictor of mortality in patients with MAC, but not in those with TB [11]. Several possible reasons can be considered. First, the patterns of disease progression differ between TB and MAC. While MAC is a chronic and slowly progressive infectious disease, TB is an acute or subacute progressive disease [22, 23]. Chronic progressive disease may decrease physical activities and affect nutritional status. Hence, these factors could influence disease progression, and vice versa. Indeed, skeletal muscle size was found to be associated with chronic respiratory diseases such as COPD and IPF [2, 3, 6, 10]. Second, effective antibacterial treatments could affect differences in impact of skeletal muscle size on mortality between TB and MAC. Patients with pulmonary TB are treated with multiple anti-TB drugs, and this condition is highly treatable. However, standard regimens including rifampicin, ethambutol, and clarithromycin can be effective in 50% of patients with MAC 1 year after sputum conversion [24]. If there are no effective treatment regimens for MAC, host factors such as muscle size are considered more influential on disease prognosis. Third, in this study, the primary outcome was in-hospital mortality. However, studies on MAC focused on lifetime mortality [11]. Long-term physical activity level or nutritional status generally declines; thus, lifetime mortality might be affected by these variables. If TB patients are followed-up after treatment completion, results may show that muscle size influences lifetime mortality. Finally, the overall ESMcsa/BSA was lower in the current research than in previous studies [5, 6, 11] probably because super-aged patients significantly accounted for the sample in this study. In Japan, newly diagnosed TB is commonly observed in elderly patients. Hence, this population might cause small variations in ESMcsa/BSA in this study.

## Limitation

The current study had several strengths. That is, it first assessed the relationship between ESMcsa/BSA and mortality in patients with TB, and all consecutive patients underwent chest CT scan. Japan is one of the countries in which physicians commonly perform chest CT scan daily. We routinely perform chest CT scan on patients with pulmonary TB to evaluate the features of this condition in detail and to rule out malignancies. However, the study also had several limitations. First, as this was a single-centered retrospective study that included a large population of elderly patients, the results are not generalizable to younger populations in other countries. However, since several countries can face aging and the aging society in the near future, these findings will be useful. Second, the primary outcome in this study was all-cause in-hospital mortality, and TB-related mortality was not distinguished from non-TB-related mortality. Whether death was associated with TB or non-TB causes is challenging to determine in clinical practice. Third, elderly patients find it difficult to maintain spine posture during chest CT scan, and this may cause measurement bias. Finally, as the ESM is a respiratory muscle, respiratory function should be investigated to evaluate its correlation with ESM/BSA. However, respiratory functional tests are not conducted from a perspective of infection control in hospitals.

In conclusion, ESM size was not associated with in-hospital mortality, whereas performance status and hemoglobin and albumin levels after adjusting for age, comorbidities, CRP level, and respiratory failure in patients with pulmonary TB. Thus, the quantification of ESM size might not be useful in predicting at least short-term prognosisin patients with pulmonary TB.

## Data Availability

All data generated or analysed during this study are included in this published article and its supplementary information files.

## Abbreviations

ESM: Erector spinae muscle
CT: Computed tomography
ESMcsa: Cross-sectional area of the ESM
BSA: Body surface area
TB: Tuberculosis
COPD: Chronic pulmonary disease
IPF: Idiopathic pulmonary fibrosis
MAC: *Mycobacterium avium* complex
BMI: Body mass index
